# Medial Femoral Condyle Flap for Nasal Support in Cocaine-Induced Midline Destruction

**DOI:** 10.1055/s-0045-1809054

**Published:** 2025-05-13

**Authors:** Pedro Alvedro-Ruiz, Belén Andresen-Lorca, Iván Heredia-Alcalde, Alessandro Thione, María Dolores Pérez-del-Caz, Alberto Pérez-García

**Affiliations:** 1 Department of Plastic and Reconstructive Surgery, University and Polytechnic Hospital La Fe, Valencia, Spain

**Keywords:** medial femoral condyle flap, nasal reconstruction, cocaine

## Abstract

Prolonged cocaine use can severely damage the osteocartilaginous structures of the midface region. Involvement of the nose, sinuses, and palate has been grouped into a syndrome called cocaine-induced midline destructive lesions. These lesions may resemble other necrotizing conditions, often complicating diagnosis and treatment. A complex nasal reconstruction is introduced in a 54-year-old woman with extensive midfacial destruction after 15 years of cocaine abuse. Total nasal reconstruction was performed using a medial femoral condyle free flap (MFCFF) for internal lining and bone support in combination with a paramedian forehead flap for external coverage. The MFCFF proved to be effective in recreating the anatomy of the nasal dorsum with minimal donor site morbidity, while the paramedian forehead flap improved the aesthetic results. Despite the need for multiple surgical interventions, this approach showed satisfactory functional and aesthetic long-term results.

## Introduction

Long-term cocaine abuse can result in progressive damage of the osteocartilaginous structures of the midface region. Destruction of nasal structures, sinuses, and palate has been grouped into a syndrome called cocaine-induced midline destructive lesions (CIMDLs). 1 These mutilating lesions may mimic other necrotizing midfacial lesions such as Wegener's granulomatosis, chronic infections, or lymphoproliferative diseases. 2

Nasal reconstruction is complex and requires an individual approach. While minor defects can be reconstructed with local flaps or composite grafts, free flaps are the most common choice for reconstruction of full-thickness defects that necessitate replacement of all layers. Several free flaps have been historically proposed, with free radial forearm free flap (RFFF) being the most commonly used. The aim of this report was to introduce a total nasal reconstruction after cocaine abuse with a medial femoral condyle free flap (MFCFF) and a paramedian forehead flap.

## Case Report



**Video 1** Intraoperative detail of the medial femoral condyle free flap (MFCFF) tailored to fit the nasal defect and flap insetting. The fatty tissue was finally not included in the flap.


**Fig. 1:**
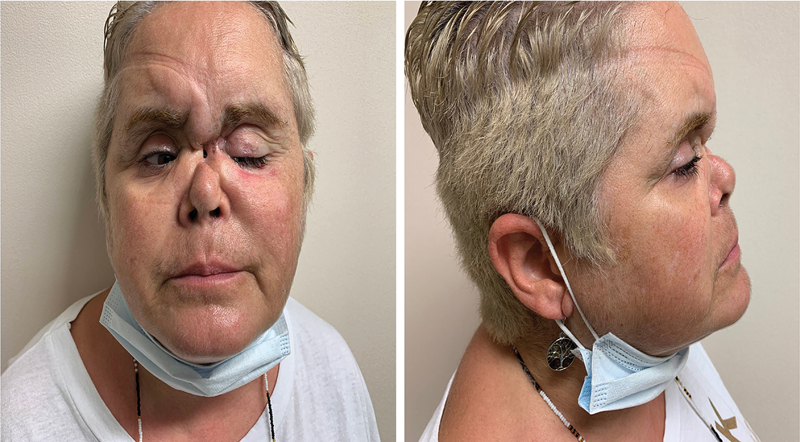
Preoperative frontal and lateral views. Notice the severe soft tissue collapse due to lack of underlying bone support.

**Fig. 2:**
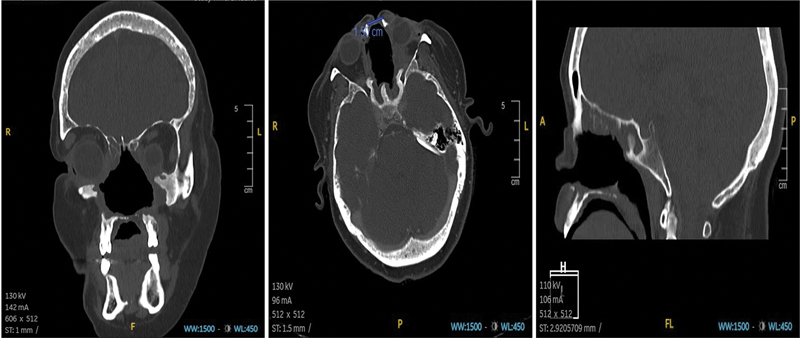
Coronal, axial, and sagittal computed tomography (CT) scans demonstrating the extensive destruction of the osteocartilaginous structures of the midface region.

A 54-year-old female was referred to our institution for nasal reconstruction evaluation. She had been taking cocaine intranasally over the past 15 years. Consequently, she exhibited extensive osteonecrosis and midface soft tissue destruction. Physical examination showed a soft tissue collapse due to the loss of the underlying bone support, with an associated nasal fistula ( Fig. 1 ). Computerized tomography (CT) scan revealed destruction of nasal structures, ethmoidal bones, maxillary and sphenoid sinuses, hard palate, and medial wall of both orbits ( Fig. 2 ). Prior to planning any reconstructive procedure, a 24-month period of abstinence from cocaine was required. A decision was made to replace the internal lining and bone support with a MFCFF and to use a paramedian forehead flap for external coverage. A two-stage reconstruction was adopted because the patient suffered from anxiety and we feared she would experience difficulties with positional instructions during the postoperative period, so we wanted to monitor the MFCFF by direct visual examination before definitive coverage.

**Fig. 3:**
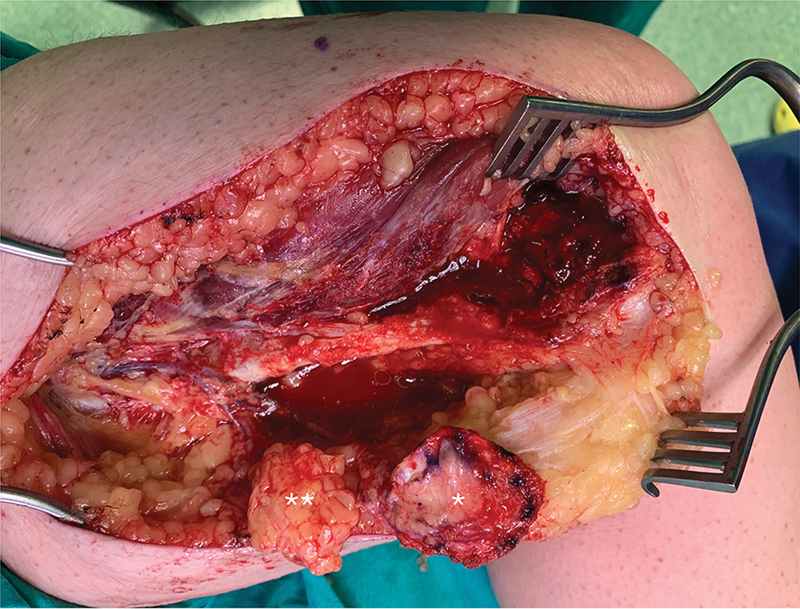
A rhomboid-shaped medial femoral condyle free flap (MFCFF) (*) was harvested from the left knee. A nearby piece of fatty tissue (**) was included in the flap with monitoring purposes.

**Fig. 4:**
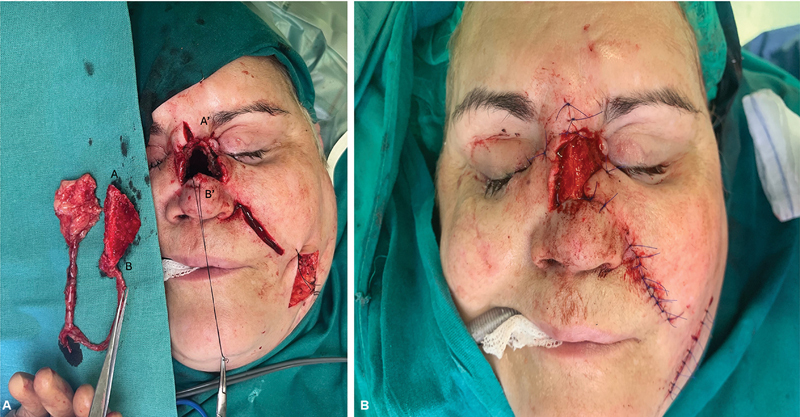
Intraoperative detail of the medial femoral condyle free flap (MFCFF) tailored to fit the nasal defect and flap insetting. The fatty tissue was finally not included in the flap. The cranial edge of the flap ( **A** ) was placed in the glabellar region (A') and the caudal edge ( **B** ) was oriented toward the nasal tip (B'). The left facial artery and vein were used as recipient vessels.

First, the midface region was debrided and the nasal tip was released from the surrounding fibrotic tissues. A 4 × 4-cm rhomboid-shaped corticoperiosteal flap was harvested as described based on the descending genicular artery and one comitant vein. A piece of fatty tissue was also raised ( Fig. 3 ). The cortical aspect of the bone was gently thinned using a bone reamer with a blunt head until it bent into the desired tent-like shape. The fatty tissue was finally not included in the inset to avoid excessive bulkiness ( Fig. 4 ). Once the flap was tailored, the periosteum was used to replace the nasal lining and the cortical aspect recreated the bony scaffold ( Video 1 ). The bone was fixed to the frontal bone and the right maxilla with 1.5-mm screws and transosseous suture, respectively. The angular vessels were first explored at the nasolabial region, but a significant caliber mismatch between the veins was observed. Microanastomosis were ultimately performed end-to-end to the left facial vessels at the level of the mandibular body. Monitoring was assessed by direct examination of bone cortical bleeding. To prevent flap desiccation moist gauzes dressings were applied twice daily, and the bone surface was irrigated with saline solution during each dressing change. Postoperative period was uneventful. The patient was discharged after 6 days.

**Fig. 5:**
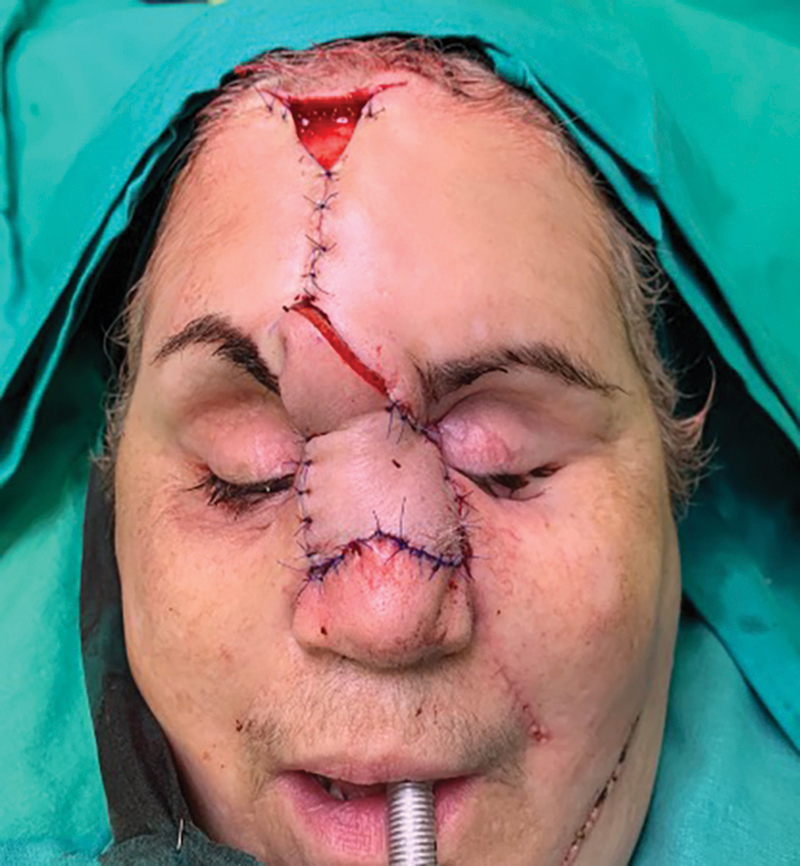
The medial femoral condyle free flap (MFCFF) was covered with a right paramedian forehead flap in a second procedure.

**Fig. 6:**
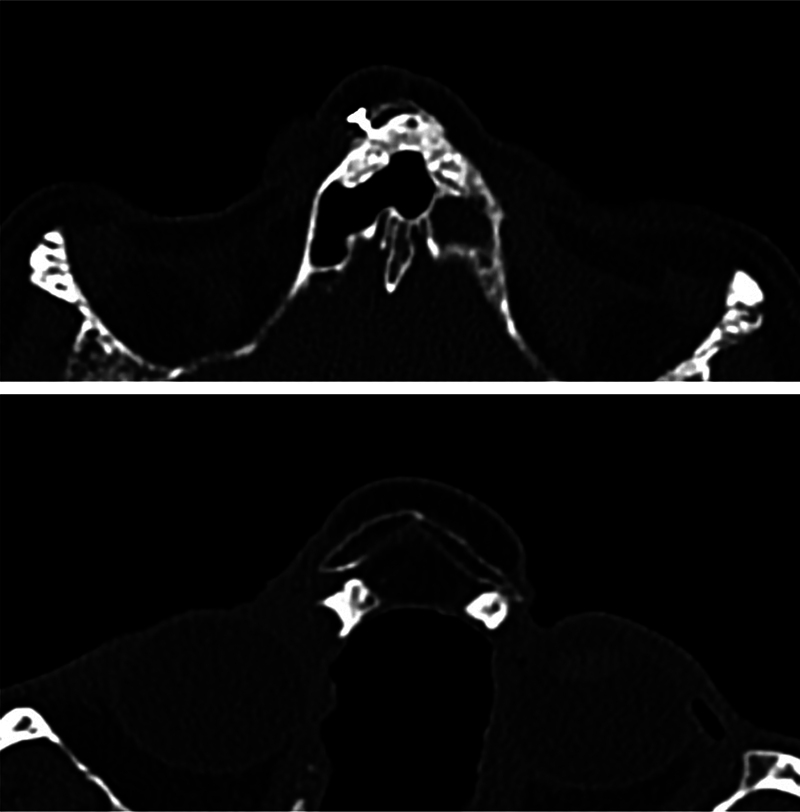
Axial computed tomography (CT) scan at 12 months of follow-up showing consolidation of the flap with the nasal spine of the frontal bone and right maxilla.

**Fig. 7:**
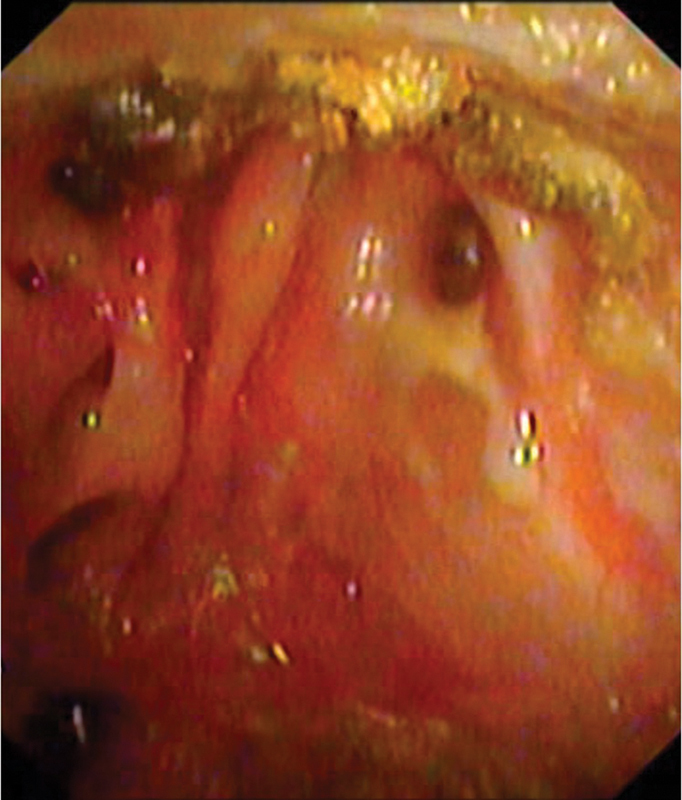
Nasal endoscopy showing periosteal mucosalization after 12 months of follow-up.

**Fig. 8:**
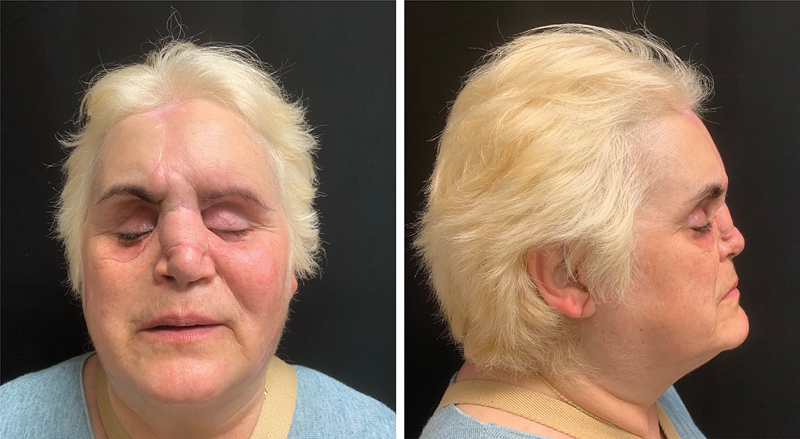
Final clinical result at 15 months of follow-up. The nasal bone support is recreated with adequate soft tissue coverage. The forehead flap scar is inconspicuous.

Two weeks later, a right paramedian forehead flap was raised to cover the MFCFF based on a template of the nasal dorsum defect ( Fig. 5 ). The flap was freed from the right supratrochlear artery and thinned 2 months later. Another procedure was performed for additional thinning. Signs of bone consolidation in both fixation points were observed at the CT scan, and nasal endoscopic evaluation confirmed periosteal mucosalization after 12 months ( Figs. 6 and 7 ). Fig. 8 shows satisfactory aesthetic outcomes after 15 months.

## Discussion

Reconstruction of total nasal defects following chronic cocaine abuse is challenging. Local skin or mucosal flaps and composite grafts are usually insufficient to deal with severe osteocartilaginous destructions. When extensive fibrotic tissues exist, nonvascularized grafts may experience long-term resorption or infections that can lead to contour deformities. In these cases, vascularized tissue transfers are mandatory to achieve acceptable functional and aesthetic results. 3 4 The RFFF is the most frequently used because of its consistent vascular supply, long pedicle, and the possibility of prelamination and harvesting it as a composite flap. 5 However, when bone scaffold is to be restored, the structural support must be recreated to assure the nasal three-dimensionality before any soft tissue coverage. First described by Sakai et al, 6 the MFCFF has been proposed as a reliable option for achieving bone union under unfavorable conditions. 7 More recently, its versatility has broadened its application to numerous anatomical regions. Gaggl et al 8 were the first to propose this flap for nasal reconstruction. Cherubino et al 9 published a case series showing the use of the MFCFF and the paramedian forehead flap for reconstruction of total nasal defects following oncologic resections. They observed good long-term outcomes and evidence of periosteal mucosalization after 6 months, reducing secondary debulking procedures. Since this corticoperiosteal flap is thin and pliable, it can be easily shaped to fit the nasal defect with conventional instruments. If the pedicle is followed to its femoral origin during the dissection, a length of 10 cm can be obtained, which is sufficient to reach the facial vessels when the flap is used for nasal or even orbital reconstruction. 10 Bone consolidation offers long-lasting support, while late resorption or infection rarely occurs. The dissection is straightforward and can be performed simultaneously with the preparation of recipient vessels by two teams. Donor site morbidity is acceptable. A paramedian forehead flap is often combined to improve the aesthetic results.

## Conclusion

Defects resulting from CIMDLs are often complex to approach and may require several flaps to achieve acceptable functional and aesthetic results. We present a case of two-stage total nasal reconstruction due to long-term cocaine abuse with a MFCFF in combination with a paramedian forehead flap. The MFCFF is a versatile and pliable corticoperiosteal flap that has been demonstrated to be a reliable option to recreate the anatomy of the nasal dorsum with minor donor site morbidity. Soft tissue reconstruction can be performed with a paramedian forehead flap.
